# Expression of individual lamins in basal cell carcinomas of the skin

**DOI:** 10.1054/bjoc.2000.1632

**Published:** 2001-02

**Authors:** R S Venables, S McLean, D Luny, E Moteleb, S Morley, R A Quinlan, E B Lane, C J Hutchison

**Affiliations:** Departments of Biological Sciences; Anatomy & Physiology; Biochemistry, University of Dundee, Dundee, DD1 5EH, UK; Department of Biological Sciences, University of Durham, South Road, Durham, DH1 3LE

**Keywords:** nuclear lamins, A-type lamins, B-type lamins, basal cell skin carcinomas

## Abstract

In this study we used a unique collection of type specific anti-lamin antibodies to study lamin expression patterns in normal human skin and in skin derived from patients with basal cell carcinomas (BCCs). Lamin expression in serial sections from frozen tissue samples was investigated by single and double indirect immunofluorescence. In normal skin, lamin A was expressed in dermal fibroblasts and in suprabasal epithelial cells but was absent from all basal epithelial cells. Lamin C was expressed in dermal fibroblasts, suprabasal epithelial cells and a majority of basal epithelial cells. However, lamin C was not expressed in quiescent basal epithelial cells. Lamin B _1_ was expressed in all epithelial cells but was not expressed in dermal fibroblasts. Finally, lamin B _2_ was expressed in all epithelial cells but was not expressed in dermal fibroblasts. Finally, lamin B _2_ was expressed in all cell types in normal skin. Lamin expression was also investigated in a collection of 16 BCCs taken from a variety of body sites. Based upon patterns of lamin expression the BCCs were classified into four groups: A-negative (10/16 tumours), C-negative (5/16 tumours), A/C-negative (1/16 tumours) and A/B _2_-negative (1/16 tumours). Lamin expression was also compared to cell proliferation index by staining serial sections with the proliferation marker Ki67. 9/10 of the lamin A negative tumours were highly proliferative, whereas 4/5 of the lamin C negative tumours were slow growing. Thus as a general rule absence of lamin A was correlated with rapid growth within the tumour, while absence of lamin C was correlated with slow growth within the tumour. Our data supports the hypothesis that lamin A has a negative influence on cell proliferation and its down regulation may be a requisite of tumour progression. © 2001 Cancer Research Campaign http://www.bjcancer.com


British Journal of Cancer (2001) 84(4), 512–519
© 2001 Cancer Research Campaign
doi: 10.1054/ bjoc.2000.1632, available online at http://www.idealibrary.com on IDEAL®

### Expression of individual lamins in basal cell carcinomas of the skin

**RS Venables[1]*, S McLean[1], D Luny[2], E Moteleb[2], S Morley[2], RA Quinlan[3], EB Lane[2] and CJ Hutchison[1*]**

Departments of [1]Biological Sciences, [2]Anatomy & Physiology and [3]Biochemistry, University of Dundee, Dundee DD1 5EH, UK; *Department of Biological Sciences, University of Durham, South Road, Durham DH1 3LE

**Summary** In this study we used a unique collection of type specific anti-lamin antibodies to study lamin expression patterns in normal human skin and in skin derived from patients with basal cell carcinomas (BCCs). Lamin expression in serial sections from frozen tissue samples was investigated by single and double indirect immunofluorescence. In normal skin, lamin A was expressed in dermal fibroblasts and in suprabasal epithelial cells but was absent from all basal epithelial cells. Lamin C was expressed in dermal fibroblasts, suprabasal epithelial cells and a majority of basal epithelial cells. However, lamin C was not expressed in quiescent basal epithelial cells. Lamin $B_1$ was expressed in all epithelial cells but was not expressed in dermal fibroblasts. Finally, lamin $B_2$ was expressed in all epithelial cells but was not expressed in dermal fibroblasts. Finally, lamin $B_2$ was expressed in all cell types in normal skin. Lamin expression was also investigated in a collection of 16 BCCs taken from a variety of body sites. Based upon patterns of lamin expression the BCCs were classified into four groups: A-negative (10/16 tumours), C-negative (5/16 tumours), A/C-negative (1/16 tumours) and A/$B_2$-negative (1/16 tumours). Lamin expression was also compared to cell proliferation index by staining serial sections with the proliferation marker Ki67. 9/10 of the lamin A negative tumours were highly proliferative, whereas 4/5 of the lamin C negative tumours were slow growing. Thus as a general rule absence of lamin A was correlated with rapid growth within the tumour, while absence of lamin C was correlated with slow growth within the tumour. Our data supports the hypothesis that lamin A has a negative influence on cell proliferation and its down regulation may be a requisite of tumour progression. © 2001 Cancer Research Campaign http://www.bjcancer.com

**Keywords**: nuclear lamins; A-type lamins; B-type lamins; basal cell skin carcinomas

The nuclear lamina is a meshwork of intermediate filaments that lines the nucleoplasmic face of the nuclear membrane and interconnects nuclear pores (reviewed by Hozak et al, 1995; Vaughan et al, 2000). Lamins, or type V intermediate filaments, consist of two subfamilies termed A-type and B-type. Whilst A and C lamins (the A-type subfamily) are alternative splice products of a single gene, there are at least four B-type genes in vertebrates (lamins $B_1$, $B_2$, $B_3$ and IV (Benavente et al, 1985a; Quinlan et al, 1995). Lamins have been ascribed a number of diverse functions including roles in the targeting of nuclear membrane vesicles to chromatin after mitosis (Burke and Gerace, 1986), correct assembly of nuclear pores (Goldberg et al, 1995) and indirectly in the organization of replication domains (Jenkins et al, 1993; Zhang et al, 1996).

A-type and B-type lamins differ in several respects including their behaviour at mitosis (Gerace and Blobel, 1980), their post-translational processing (Farnsworth et al, 1989) and their expression during differentiation. B-type lamins are ubiquitous components of all cells, although different cells do express different B-type lamins (Stick and Hausen, 1985; Furukawa and Hotta, 1993). The two most prominent B-type lamins in mammalian somatic cells are lamins $B_1$ and B2, although not all somatic tissues express both of these B-type lamins (Benavente and Krohne, 1985b; Stick and Hausen, 1985; Lehner et al, 1987; Broers et al, 1997).

*Received 6 June 2000*
*Revised 9 November 2000*
*Accepted 14 November 2000*

*Correspondence to:* EB Lane/CJ Hutchison

The expression of A-type lamins appears to be linked to differentiation and is first observed in embryos at the time of organogenesis (Schatten et al, 1985; Stewart and Burke, 1987; Röber et al, 1989). Pluripotent cells can be induced to express A-type lamins in culture by treatment that induces differentiation (Lebel et al, 1987) and in some instances the ectopic expression of lamin A can promote differentiation (Lourim and Lin, 1992). The correlation between A-type lamin expression and cell differentiation has led some workers to speculate that A-type lamins may facilitate differential gene expression, by anchoring chromatin at the nuclear envelope or by sequestering inhibitors (Nigg, 1989). Consistent with this hypothesis, lamin A displays a higher affinity for chromatin binding than B-type lamins (Höger et al, 1991). A-type lamins also bind the negative growth regulator $p110^{RB}$ (Ozaki et al, 1994).

The expression of A-type lamins is also altered in many tumours. In lung tumours there is an inverse correlation between the level of the proliferation marker Ki67 staining with that of the anti-lamin A/C monoclonal BU31 (Rowlands et al, 1994). Changes in the expression of lamin A has also been reported in testicular germ cell tumours (Machiels et al, 1997) and Hodgkins disease (Jansen et al, 1997). In addition, adrenal cortex carcinoma cell lines and certain lung adenocarcinoma cell lines display marked imbalances between the expression of lamins A and C (Paulin-Levasseur et al, 1988; Machiels et al, 1995). The fast growing small cell lung carcinomas also do not express A-type lamins whereas non-small cell lung carcinomas do (Broers and Ramaekers, 1994) reinforcing the suggestion of a relationship between expression of A type lamins and growth rate and differentiation status.

In this study we have used an extensive and unique panel of type-specific lamin antibodies to study lamin expression in human skin to look further at lamin expression in normal and diseased tissue. Our results reveal different expression patterns, not only between A-type and B-type lamins, but also between lamins A and C and between lamins $B_1$ and $B_2$ in different cell types. Furthermore, we report that while absence of lamin A is characteristic of basal cell skin carcinomas (BCCs), absence of lamin C occurs in only a sub-set of BCCs.

### MATERIALS AND METHODS

### Antibodies

A newly-generated antibody specific for lamin C was used in this study. This rabbit antiserum was raised against the last 8 amino acids of lamin C, including an N-terminal lysine as a linker (KHHVSGSRR). The peptide was coupled to keyhole limpet haemocyanin through primary amino groups using glutaraldehyde. The resulting protein-peptide conjugate was dialysed overnight at 4˚C against PBS. The conjugate was then used to immunize a rabbit. Immune serum was screened by indirect immunofluorescence and then affinity purified against 10 mg of the lamin C peptide conjugated to CH Sepharose 4B as described in Harlow and Lane (1988).

All the primary antibodies to lamins used in this study are listed in Table 1, together with their sources and specificities. Rabbit antiserum to Ki67 was purchased from DAKO and used at a dilution of 1:50. All secondary antibodies were obtained commercially: FITC- and peroxidase-conjugated goat anti-mouse IgG, TRITC- and peroxidase-conjugated donkey anti-goat IgG and FITC-, TRITC- and peroxidase-conjugated sheep anti-rabbit IgG were all purchased from DAKO and used according to the manufacturer's instructions. All dilutions were done in phosphate buffered saline (PBS) containing 1% newborn calf serum.

### Immunofluorescence

HeLa epithelial cells or human dermal fibroblasts (HDF) were seeded at an initial density of $2 \times 10^5$ on 90 mm plates containing 13 mm diameter glass coverslips. The cells were grown to approximately 80% confluence in DMEM supplemented with 2 mM glutamine, 10 U $ml^{-1}$ penicillin, 100 µg $ml^{-1}$ streptomycin, plus 10% newborn (for HeLa cells) or fetal (for HDF cells) bovine serum. The cells were then fixed in methanol/acetone (1:1 v/v) for 10 minutes at 4˚C and washed 3 times in PBS before immunostaining.

Biopsies of normal skin from different body sites (surgical trimmings from groin, dorsal-proximal finger, face and back areas) and of basal cell carcinomas (BCCs) from 16 individual patients were snap-frozen in liquid nitrogen. Frozen blocks were sectioned on a Reichert Jung cryomicrotome to give serial sections of approximately 6–7 µm thickness. Sections were collected on uncoated microscope slides and stored at –70˚C. Prior to use they were removed from the freezer, thawed and dried rapidly in a stream of warm air. All staining procedures were then carried out at room temperature. The sections were incubated with 1% fetal calf serum in phosphate buffered saline (PBS) for 10 minutes to block non-specific protein binding, then rinsed in PBS for 5 minutes.

For immunofluorescence of cells or tissue sections, antibody incubations were all for 1 h at room temperature in a humidified environment, followed by 5 min wash in PBS. After the secondary antibody and wash, a final wash in water was used before mounting. Specimens were mounted in Mowiol containing 1 µg $ml^{-1}$ DAPI (4´,6-diamidin-2-phenylindol-dihydrochloride) and 1 µg $ml^{-1}$ DABCO (1,4-diazabicyclo[2.2.2]octane). Immunofluorescence samples were examined with a Zeiss Axiovert 10 microscope equipped for epifluorescence using × 63 N/A 1.4 and × 40 N/A 1.3 PlanNeofluar lenses. Images were collected with a 12 bit CCD camera using IPLab software.

**Table 1** Lamin-specific antibodies used in this study

| Antibody | Lamin target | Antibody type | Dilution used | Source |
|---|---|---|---|---|
| 133A2 | Lamin A | Mouse/m | 1:1000 | Hozak et al, 1995 |
| JoL4 | Lamin A | Mouse/m | Undiluted | Dyer et al, 1999 |
| JoL2 | Lamin A/C | Mouse/m | Undiluted | Dyer et al, 1999 |
| RaLC | Lamin C | Rabbit/p | 1:50 | This study |
| GaLB1 | Lamin B1 | Goat/p | 1:20 | Santa Cruz |
| LN43 | Lamin B2 | Mouse/m | 1:100 | Dyer et al, 1999 |

p = polyclonal antiserum, affinity purified; m = monoclonal antibody.

### Immunoblotting for antibody characterization

Three fragments of the lamin C gene encoding residues 1–171aa, 171–319aa and 319–572aa were cloned into pGEX expression vectors and expressed in *E. coli* strain BL21 and purified according to previously published protocols (Dyer et al, 1997; Pugh et al, 1997). Nuclear matrix preparations of HeLa cells were performed following previously published protocols of Mattern et al (1992)

Samples prepared for SDS-PAGE were resolved on a 12% polyacrylamide gel. Immunoblotting was carried out as described by Jenkins et al (1993) using the following primary antibodies (see Table 1): rabbit anti-lamin C (to lamin C) at 1:1000, JoL2 (to lamins A,C) used undiluted, JoL4 (to lamin A) used undiluted, LN43 (to lamin $B_2$) diluted to 1:1000 and affinity purified goat anti-lamin B1 used at 1:500 (Santa Cruz). Detection of the primary antibody used antibodies conjugated to horseradish peroxidase as above. All antibody incubations were for 1 hour at room temperature. The blots were developed using the ECL detection method.

### RESULTS

### Confirmation of antibody specificities

The aim of this study was to distinguish between the expression and distribution of lamin subtypes and an essential first step was therefore to confirm the antibody specificities. On immunoblotting, the rabbit antiserum reacted strongly with fragments corresponding to peptide 319–572aa of lamin C but with no other fragment (Figure 1A). When tested against nuclear protein extracts from HeLa cells monoclonal antibodies specific for lamin A (JoL4 – Figure 1B, also 133A2) reacted with a single band migrating at 70 kDa. JoL2 against lamins A and C, reacted with two bands migrating at 70 kDa and 65 kDa respectively (Figure 1B, lane 1). The anti-peptide antiserum to lamin C reacted with a single band migrating at 65 kDa (Figure 1B, lane 3). The goat polyclonal antibody against lamin $B_1$ reacted with a single band migrating at 67 kDa (Figure 1B, lane 4). Finally, the monoclonal antibody against lamin $B_2$ (LN43) reacted with a single band migrating slightly behind the lamin B band at 68 kDa (Figure 1B, lane 5). The expected mobilities of human lamins A, C, $B_1$ and $B_2$

© 2001 Cancer Research Campaign

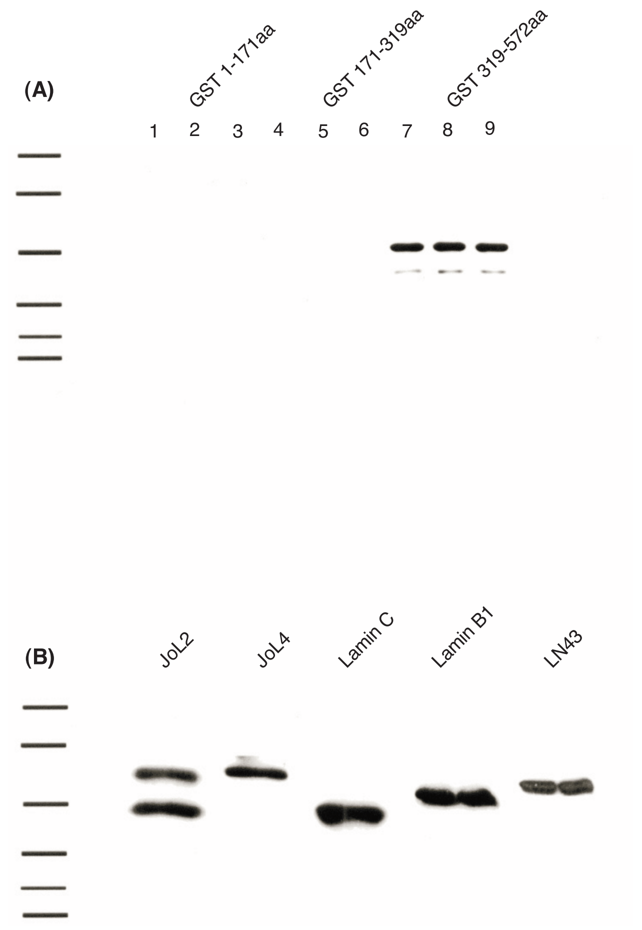

**Figure 1** Characterization of type specific lamin antibodies by immunoblotting. (**A**) GST fusion proteins were used to confirm the specificity of rabbit anti-lamin C antibody. GST-fusion peptides spanning the following residues of the lamin C gene, 1–171aa (lanes 1–3), 171–319aa (lanes 4–6) and 319–572aa (lanes 7–9), were expressed in *E. coli* and purified. The purified fragments were resolved on SDS-PAGE, transferred to nitrocellulose and blotted with the anti-lamin C antibody. The antibody reacted exclusively with the fusion protein containing the C-terminal fragment 319–572aa that contains the 6 amino acids specific to lamin C. (**B**) Nuclear matrix fractions from Hela cells were resolved on SDS-PAGE, transferred to nitrocellulose and immunoblotted with one of mAb anti-lamin A/C – JoL2, mAb anti-lamin A – JoL4, goat anti-lamin B1, mAb anti-lamin B2 – LN43 or rabbit anti-lamin C. Molecular weight markers = 175, 83, 62, 47.5, 32.5, 25, 16

on SDS PAGE are 70 kDa, 65 kDa, 67 kDa and 68 kDa respectively, thus the results of immunoblotting support the specificities of the antibodies. Since the mobilities of lamin $B_1$ and lamin $B_2$ are very close, we wished to confirm that the commercial lamin $B_1$ antibody reacted with lamin $B_1$. Therefore, LN43 and the goat polyclonal antibodies were both used to blot recombinant human lamin $B_1$. As expected LN43 did not react with recombinant lamin $B_1$ whereas the goat polyclonal antibody reacted strongly (data not shown).

Indirect immunofluorescence was performed on HeLa cells (Figure 2) and HDF (not shown). JoL2 (Figure 2A), LN43 (Figure 2E), goat anti-lamin $B_1$ (Figure 2D) and rabbit anti-lamin C (Figure 2C) all gave rise to pronounced 'rim' staining in HeLa cells and proliferating HDF, typical of localisation to the nuclear lamina. In contrast, JoL4 stained both the nuclear rim and intranuclear speckles in both cell types (Figure 2B). Taken together these data confirm the lamin specificities indicated in Table 1 and especially that the rabbit polyclonal antibody generated for these studies specifically reacts with lamin C only.

### Distribution of lamins in normal human skin

Immunofluorescence on frozen unfixed sections of skin from different body sites gave consistent results for each lamin. Typical examples (all from finger skin) are shown in Figure 3. Two different monoclonal antibodies against lamin A (JoL4 and 133A2) stained dermal fibroblasts and suprabasal epidermal cell nuclear envelopes intensely, but basal epidermal cells were mostly unstained (arrows Figure 3 D–E, Figure 4A). In contrast, JoL2 (anti-lamin A/C) stained dermal fibroblasts, suprabasal epidermal cells and the majority of epidermal basal cells. It was noticeable that a population of the basal epidermal cells were not stained by lamin A/C antibodies (Figure 3A–C; Figure 4C).

The goat anti-lamin $B_1$ antibody stained suprabasal and basal epidermal cells uniformly. In contrast, dermal fibroblasts were mostly unstained (arrows, Figure 3G–I). The lamin $B_2$ antibody LN43 stained all cell types in human skin (Figure 3J–L). The rabbit polyclonal antibody against lamin C (RαLC) gave rise to a staining pattern that was almost identical to JoL2 (anti-lamin A/C): dermal fibroblasts, suprabasal epidermal cells and a majority of basal epidermal cells were all stained. However, a small number of basal epidermal cells were unstained (arrows Figure 3 M–O).

Taken together, these data indicate differences in the expression of the lamin sub-types in different cell types within the epidermis. Lamin $B_2$ is expressed uniformly in all nucleated cells within human skin (epidermis and dermis). Lamin $B_1$ is expressed throughout the epidermis but as reported previously (Broers et al, 1997) was not detected in dermal fibroblasts. Lamin A appears to be expressed in dermal fibroblasts and in suprabasal epidermal layers, but most cells, in the basal layer did not express lamin A. Lamin C is also expressed in all dermal fibroblasts and in a majority of epidermal cells, but lamin C was still not detected in a small number of epidermal basal cells. Since JoL2 (A/C) and RαLC give identical staining patterns (this was confirmed by double immunofluorescence, not shown), but very different patterns from those observed with JoL4 and 133A2 (A), we infer that staining of basal epidermal cells with JoL2 indicates the presence of lamin C.

### Differences in the expression of lamin A and lamin C in epidermal basal cells

In the epidermal basal cell layer, lamin A is not expressed in the majority of cells, whereas lamin C is absent from approximately 1 in every 40 cells. To investigate this phenomenon further, double indirect immunofluorescence was performed with monoclonal antibodies JoL2, JoL4, 133A2 and a rabbit polyclonal antibody against the proliferation marker Ki67. Whilst again 133A2 and JoL4 staining (lamin A) was absent from both Ki67-negative and Ki67-positive cells (Figure 4A, B) in the basal epidermis, JoL2 (A/C) staining was present in and apparently restricted to all Ki67-positive basal cells (arrowheads Figure 4C, D). These data suggest that A-type lamins are not expressed in a subpopulation of quiescent non-cycling basal cells in the epidermis, but that lamin C, but not lamin A, is up-regulated in cells that are stimulated to divide. Lamin A expression appears later in the process of differentiation.

 © 2001 Cancer Research Campaign

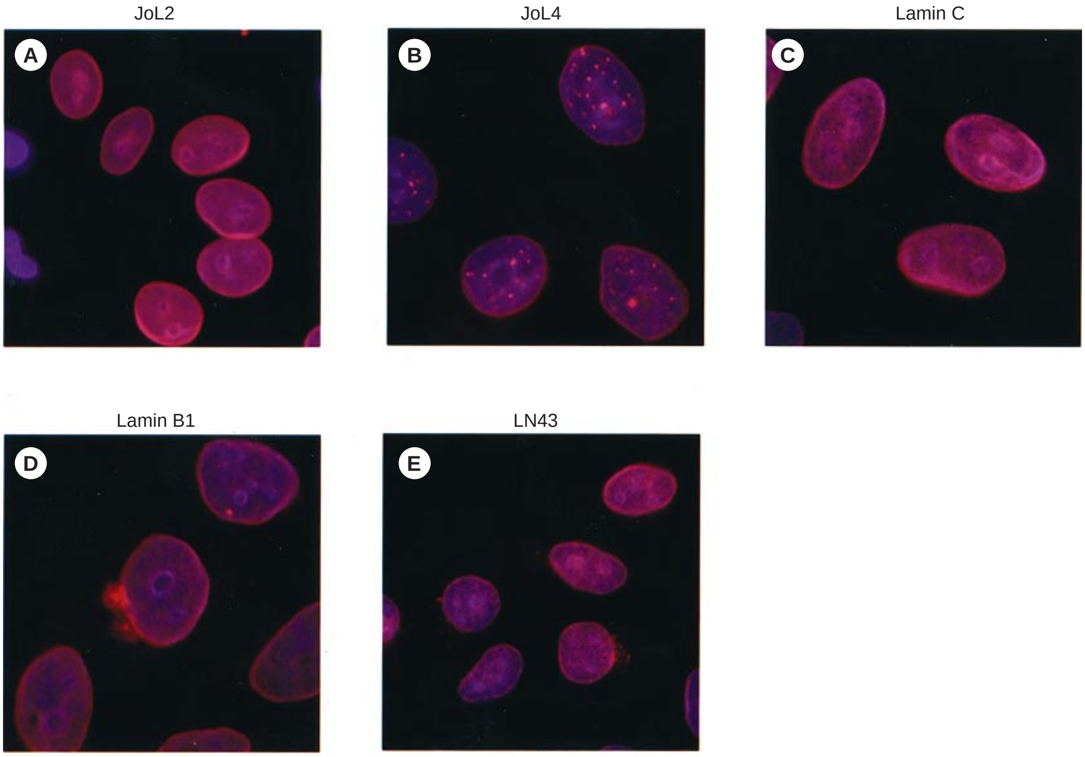

**Figure 2** Characterization of type specific anti-lamin antibodies by immunofluorescence. HeLa cells were fixed with methanol:acetone and stained with one of monoclonal antibody anti-lamin A/C JoL2 (**A**), monoclonal antibody anti-lamin A JoL4 (**B**), rabbit anti-lamin C (**C**), goat anti-lamin B1 (**D**) and mAb anti-lamin B2 LN43 (**E**). In each sample cells were co-stained with DAPI to reveal the distribution of chromatin. Each micrograph represents a merged image in which the antibody staining (in red) is superimposed over the DAPI stain (in blue). All lamin antibodies displayed a characteristic nuclear rim staining except JoL 4 that stained both the nuclear rim and intranuclear foci. Scale bars = 10 μm

### Changes in the expression of lamins A and C in basal cell skin carcinomas

Changes in the expression of A-type lamins have been reported in a range of tumours. We investigated lamin expression in a collection of 16 basal cell skin carcinomas (BCC) taken from a variety of body sites including back, elbow and neck. In each instance lamin expression in normal (peri-tumoral) tissue and tumour lobes was compared. Typical results are displayed in Figures 6 and 7 while a summary of results is displayed in Table 2. All tumours retained expression of lamin $B_1$, and all except one expressed lamin $B_2$ also. The A-type lamins were more variable however. Most tumours expressed either lamin A (detected with JoL4 and 133A2) or lamin C but not both, although one tumour had neither. The tumours could therefore be divided up into four groups on the basis of their lamin expression: A-negative (10/16 tumours), C-negative (5/16 tumours), A/C-negative (1/16 tumours) and A/$B_2$-negative (1/16 tumours). Finally, in one tumour from the A-negative group, lamin C was expressed but it was localized in the nucleolus rather than in the nuclear envelope (Figure 5; Table 2).

To determine whether lamin expression was correlated with rate of growth within the tumour, serial sections from the same tumours were also stained for Ki67 expression. Of the A-negative tumours, 9 out of 10 were highly proliferative as indicated by extensive Ki67 staining within the tumour lobe (Table 2). In

**Table 2** Expression of individual lamins and Ki67 antigen in basal cell carcinomas of the skin

| Patient | Lamin A | Lamin A/C | Lamin C | Lamin B1 | Lamin B2 | Ki67 |
|---|---|---|---|---|---|---|
| 1. | ++ | ++ | ++ | ++ | ++ | ++ |
| 2. | ++ | ++ | ++ | ++ | ++ | – |
| 3. | – | + | + | ++ | ++ | ++ |
| 4. | – | + | + | ++ | +++ | +++ |
| 5. | – | + | + | ++ | ++ | +++ |
| 6. | +/– | + | ++ | ++ | ++ | ++ |
| 7. | – | ++ | ++ | ++ | +/– | ++ |
| 8. | +/– | ++ | ++ | +++ | ++ | +++ |
| 9. | +/– | ++ | ++ | ++ | +++ | +++ |
| 10. | – | ++ | ++ | ++ | ++ | ++ |
| 11. | – | +/– | + nucleolar | ++ | ++ | +/– |
| 12. | – | – | – | ++ | +++ | ++ |
| 13. | + | + | – | ++ | ++ | +/– |
| 14. | + | + | – | ++ | ++ | +/– |
| 15. | + | + | – | +++ | +++ | +/– |
| 16. | ++ | ++ | – | +++ | ++ | – |

Frozen sections of basal cell skin carcinomas were stained individually with type specific anti-lamin antibodies or with rabbit ant-Ki67. The level of expression of each antigen within tumour lobes was scored according to an arbitrary scale in which – indicates low level or absent staining while +++ indicates maximum staining. +/– indicates some positive some negative. Patient numbers are indicated in the left hand column. Nucleolar (lamin C patient 96/96) indicates that in this section anti-lamin C antibodies stained the nucleolus.

*© 2001 Cancer Research Campaign* 

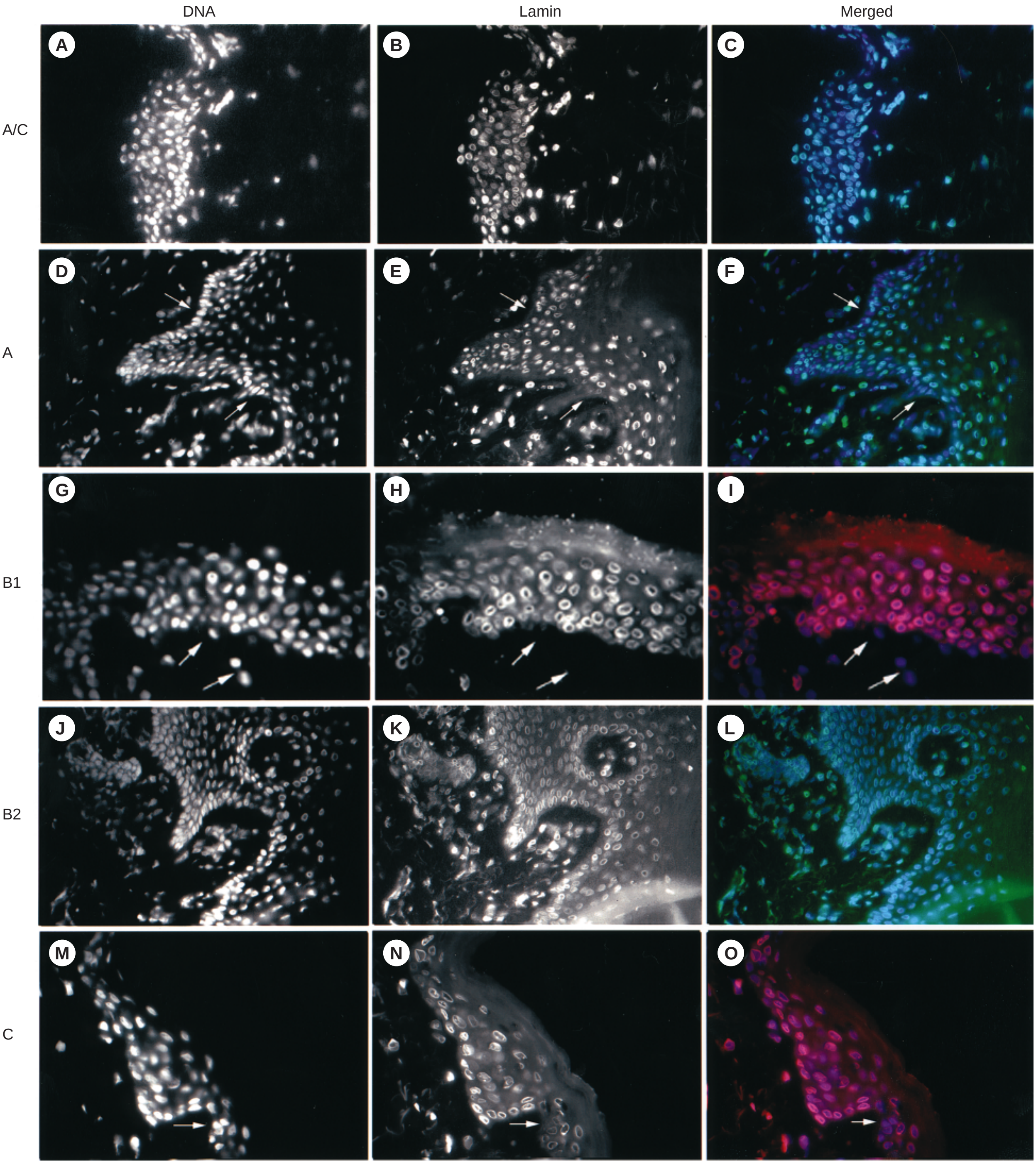

**Figure 3** Lamin expression in normal human finger skin. Frozen sections of normal human finger were stained with either JoL2 (anti-lamin A/C – panels A–C), 133A2 (anti-lamin A – panels D–F), goat anti-lamin B1 (panels G–I), LN43 (anti-lamin B2–panels J–K) or rabbit anti-lamin C (panels M–O). Sections were co-stained with DAPI to reveal nuclei. Panels A, D, G, J, M show DAPI stained images. Panels B, E, H, K and n show antibody stained images. Panels C, F, I,L and O show merged images. Arrows illustrate representative nuclei in each section that are unstained. Scale bars = 100 μm contrast, 4 out of the 5 C-negative tumours were slow growing as indicated by an absence of Ki67 staining within the tumour lobe (Table 2). Therefore as a general rule, absence of lamin A alone was correlated with rapid growth within the tumour, whereas absence of lamin C was correlated with slow growth within the tumour.

### DISCUSSION

Until now there have been very few attempts to functionally discriminate between lamins A and C, largely because of the difficulty in doing so. The generation of an antibody specific to lamin C now allows a re-examination of the expression of A-type

 © 2001 Cancer Research Campaign

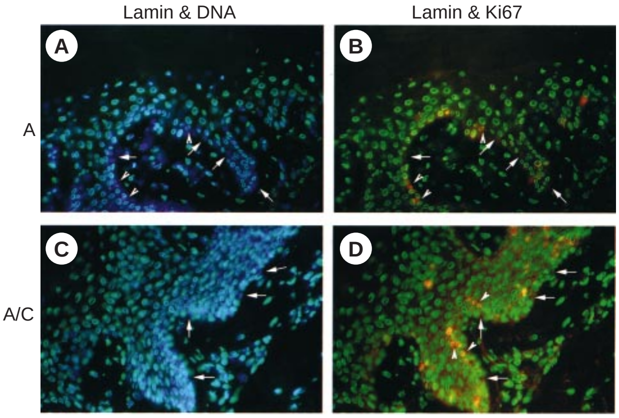

**Figure 4** Lamin A and C expression in basal epidermis. Frozen sections of finger skin were co-stained with JoL2 (anti-lamin A/C), Ki67 and DAPI (panels A and B) or JoL4 (anti-lamin A), Ki67 and DAPI (panels C and D). Each panel represents a two colour merged image in which the anti-lamin antibody stain (in green) is superimposed over the DAPI stain (in blue – panels A and C) or the Ki67 stain (in red – panels B and D). Arrows indicate cells that are Ki67 negative: arrowheads indicate cells that are Ki67 positive. Scale bars = 100 μm lamins in normal and cancerous tissue. In this investigation, the absence of expression of one or other A-type lamin in the majority of a group of 16 basal cell carcinomas of the skin has been shown (the A-negative BCC type). Most tumours show a down-regulation of lamin A expression, while a smaller group of tumours (C-negative) gave negative results for lamin C expression. In one tumour neither A nor C lamins were detected. In another tumour, absence of lamin $B_2$ expression accompanied absence of lamin A expression. In a third BCC, lamin C was present but abnormally located in the nucleolus.

Down regulation of A-type lamin expression is a feature of a number of tumour types including small cell lung carcinomas (Broers and Ramaekers, 1994), testicular cancer (Machiels et al, 1997) and Hodgkins disease (Jansen et al, 1997). In lung cancer, changes in the level of lamin expression (Broers et al, 1993) have been linked to expression of v-rasH (Kaufman et al, 1991), but there is apparently no common mechanism by which down regulation of lamin expression occurs in tumours. The lamin A gene has been mapped to 1q12.1-q23, a region of chromosome 1 that is commonly rearranged in tumours (Kamat et al, 1993). Gene deletion (Kamat et al, 1993) as well as transcriptional and

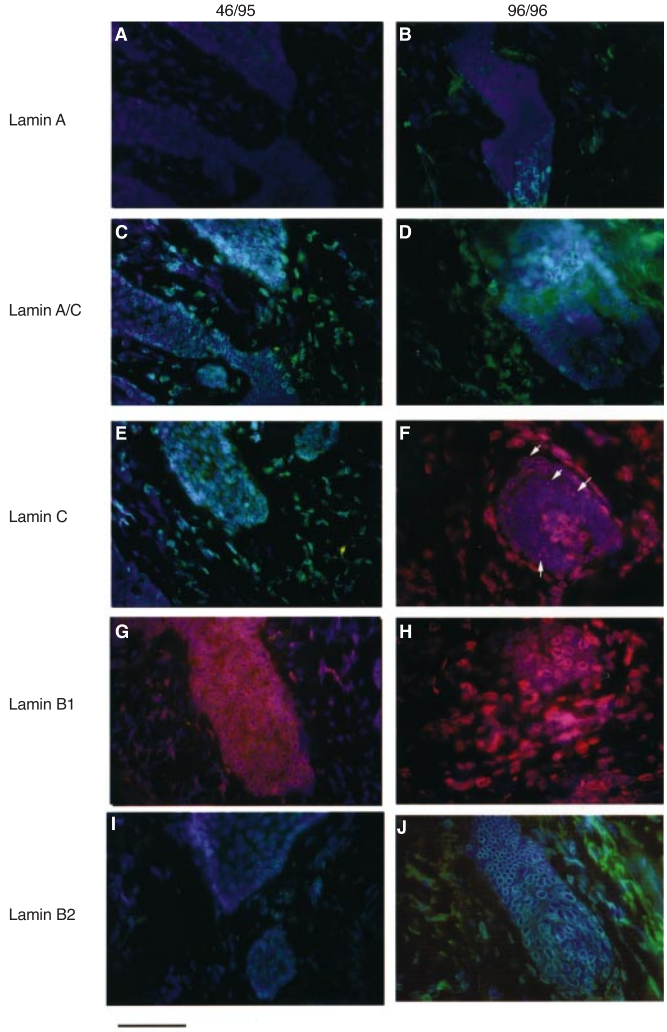

**Figure 5** Lamin expression in basal cell skin carcinomas. Frozen sections from BCCs were co-stained with one of JoL 4 (anti-lamin A – panels A and B), JoL 2 (anti-lamins A/C – panels C and D), rabbit anti-lamin C (panelsE and F), goat anti-lamin B1 (panels G and H) or LN43 (anti-lamin B2 – panels I and J) and DAPI. In each panel the antibody stain (in red or green) is superimposed over the DAPI image (in blue). The left hand panels show staining of patient number 46/95 that is a representative lamin A negative, lamin C positive tumour. The right hand panels show an atypical lamin A negative, lamin C positive tumour (number 96/96) in which lamin C is localized in the nucleolus (arrows panel F). Scale bars = 100 μm

© 2001 Cancer Research Campaign

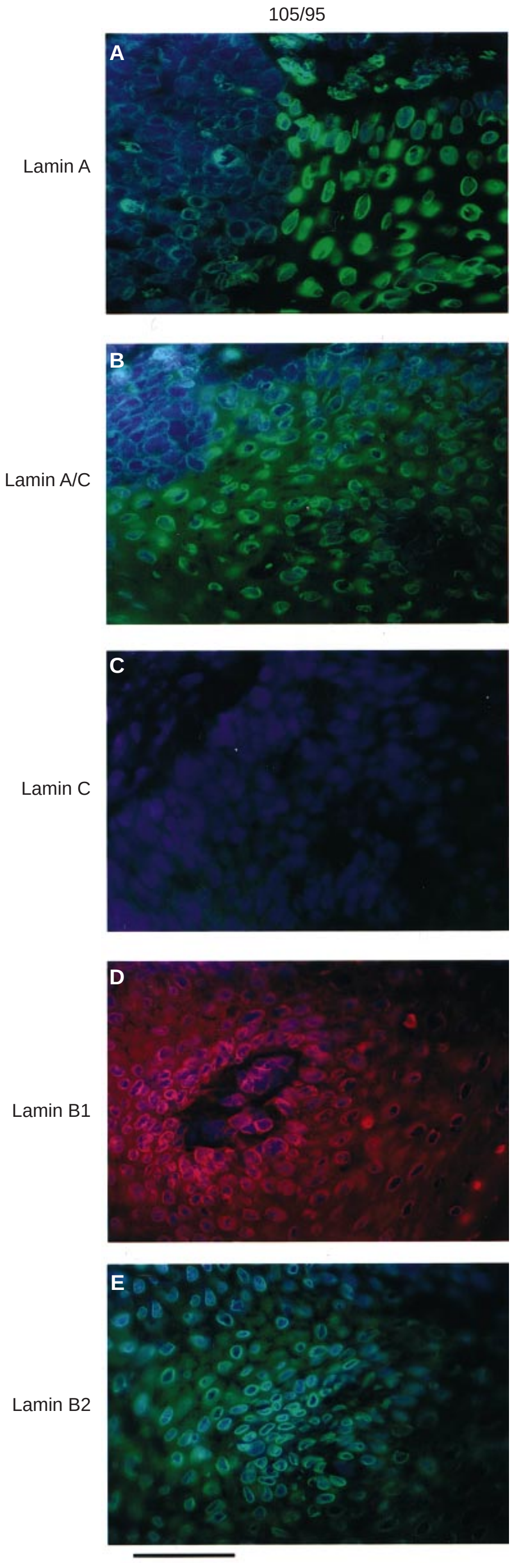

**Figure 6** Lamin expression in basal cell skin carcinomas. Frozen sections from BCCs were co-stained with one of JoL4 (anti-lamin A – panel a), JoL2 (anti-lamin A/C – panel b), rabbit anti-lamin C (panel c), goat anti-lamin B1 (panel d) or LN43 (anti-lamin B2 – panel e) and DAPI. In each panel the antibody stain (in red or green) is superimposed over the DAPI image (in blue). The figure illustrates a representative lamin C-negative tumour. Scale bars = 100 μm
p = polyclonal antiserum, affinity purified; m = monoclonal antibody.

post-transcriptional mechanisms (Lebel et al, 1987; Machiels et al, 1996) have all been implicated in the loss of lamin A or C expression. Therefore an important question is whether changes in lamin expression reflect de-differentiation within the tumour or is a requisite of tumour progression.

10 out of the 16 basal cell carcinoma tumour samples included in this investigation did not show lamin A expression. Lamin A was not detected in most epidermal basal cells in normal tissue, suggesting that these cells could be the originators of the A-negative tumours. Thus the lamin A detected in 6 samples could reflect up-regulation in these tumours. Finally, 5 samples showed an absence of lamin C in tumour lobes, although lamin C was expressed in normal proliferating basal cells of the epidermis, suggesting that this could reflect downregulation of lamin C expression. These data therefore suggest a complex pattern of lamin A/C expression that is not controlled by a single mechanism. In the majority of cases, absence of lamin A might be explained by the origin of the tumour (i.e. from lamin-A negative basal epidermal cells). However previous studies have shown that expression of lamin A is down-regulated as mouse fibroblasts progress from a quiescent to a proliferating state (Pugh et al, 1997). The majority of tumours failing to stain with lamin A antibodies were hyperproliferative, as seen by Ki67 staining, and an alternative explanation for the absence of lamin A in these tumours is through cell-cycle dependent mechanisms. Tumours displaying absence of lamin C were generally slower growing (less Ki67). Changes in lamin C expression resulting from changes in cell cycle status have not been detected in earlier studies (Dyer et al, 1997; Pugh et al, 1997). Therefore, the absence of lamin C in slow-growing tumours was somewhat surprising but again indicates underlying selective changes in lamin expression associated with the rate of growth in the tumour. Since lamin A and lamin C are alternatively spliced variants of the same gene (Lin and Worman, 1993), the differences in expression of these two lamins must arise through post-transcriptional mechanisms (Lanoix et al, 1992). The availability of specific antibodies against lamin C allows for further study of this phenomenon which will be essential in order to understand why the expression of these two lamins is linked to the rate of tumour growth.

Lamin A has been reported as an in vitro binding partner for the tumour suppressor protein p110$^{RB}$ (Ozaki et al, 1994), and p110$^{RB}$ is tightly associated with nuclear substructures in its hypophosphorylated form (Templeton et al, 1991; Mancini et al, 1997). Although lamins are normally distributed at the nuclear envelope, more recent evidence has shown that lamin A is also located at a number of intranuclear sites (Goldman et al, 1992; Bridger et al, 1993), which could provide nuclear anchorage sites for p110$^{RB}$. If as suggested, nuclear anchorage is important for the function of p110$^{RB}$ in transcriptional silencing (Mittnacht and Weinberg, 1991), an absence of lamin A may favour unregulated cell division. Therefore, its presence or absence may directly influence the proliferative status of a tumour. That lamin A is absent in hyperproliferative basal cell skin carcinomas supports this hypothesis.

### ACKNOWLEDGEMENTS

This work was generously supported by the Cancer Research Campaign (SP2060/0102 to EBL and SP2101/0401 to CJH, RAQ, EBL)

 *© 2001 Cancer Research Campaign*

### REFERENCES

Benavente R and Krohne G (1985a) Change of karyoskeleton during spermatogenesis of *Xenopus*: Expression of lamin $L_{iv}$, a nuclear lamina protein specific for the male germline. *Proc Nat Acad Sci* **82**: 6176–6180

Benavente R, Krohne G and Franke W (1985b) Cell type specific expression of nuclear lamina proteins during development of *Xenopus laevis*. *Cell* **41**: 177–190

Bridger JM, Kill IR, O'Farrell M and Hutchison CJ (1993) Internal lamin structures within the G1 nuclei of human dermal fibroblasts. *J Cell Science* **104**: 297–308

Broers JLV and Ramaekers FCS (1994) Differential markers for lung-cancer sub-types. A comparative study of their expression *in vivo* and *in vitro Int J Cancer Suppl* **8**: 134–137

Broers JLV, Carney DN, Klein Rot M, Kuijpers H, Wagenaar S and Ramaekers FCS (1993) Nuclear A-type lamins are differentially expressed in human lung cancer subtypes. *Am J Pathol* **143**: 211–220

Broers JLV, Machiels, BM, Kuijpers HJH, Smedts, F, Van den Kieboom R, Raymond, Y and Ramaekers FCS (1997) A and B-type lamins are differentially expressed in normal human tissues. *Hist Chem Cell Biol* **107**: 505–517

Burke B and Gerace L (1986) A cell free system to study reassembly of the nuclear envelope at the end of mitosis. *Cell* **44**: 639–652

Dyer JA, Kill IR, Pugh G, Quinlan RA, Lane EB and Hutchison CJ (1997) Cell cycle changes in A-type lamin associations detected in human dermal fibroblasts using monoclonal antibodies. *Chrom Res* **5**: 383–394

Dyer JA, Lane EB and Hutchison CJ (1999) Investigations of the pathway of incorporation and function of lamin A in the nuclear lamina. *Microsc Res Tech* **45**: 1–12

Farnsworth C, Wolda S, Gelb M and Glomset J (1989) Human lamin B contains a farnsylated cysteine residue. *J Biol Chem* **264**: 20422–20429

Furukawa K and Hotta Y (1993) cDNA cloning of a germ cell specific lamin $B_3$ from mouse spermatocytes and analysis of its function by ectopic expression in somatic cells. *EMBO J* **12**: 97–106

Gerace L and Blobel G (1980) The nuclear envelope lamina is reversibly polymerised during mitosis. *Cell* **19**: 277–287

Goldberg M, Jenkins HE, Allen T, Whitfield WGF and Hutchison CJ (1995) *Xenopus* lamin $B_3$ has a direct role in the assembly of a replication competent nucleus: evidence from cell-free egg extracts. *J Cell Sci* **108**: 3451–3461

Goldman AE, Moir RD, Montaglowy M, Stewart M and Goldman RD (1992) Pathway of incorporation of micro-injected lamin A into the nuclear envelope. *J Cell Biol* **119**: 725–735

Harlow E and Lane DP (1988) *Antibodies: A laboratory manual*. Cold Spring Harbor Laboratory: NY

Höger T, Krohne G and Kleinschmidt JA (1991) Interaction of *Xenopus* lamins A and Lii with chromatin *in vitro* mediated by a sequence in the C-terminal domain. *Exp Cell Res* **197**: 280–289

Hozak P, Sasseville A, Raymond Y and Cook PR (1995) Lamin proteins form an internal nucleoskeleton as well as a peripheral lamina. *J Cell Sci* **108**: 635–644

Jansen MPHM, Machiels BM, Hopman AHN, Broers JLV, Bot FJ, Arends JW, Ramaekers FCS and Schouten HC (1997) Comparison of A- and B-type lamin expression in nodular sclerosing Hodgkin's disease and reactive lymph nodes. *Histopathology* **4**: 304–312

Jenkins HE, Holleman T, Lyon C, Lane EB, Stick R and Hutchison CJ (1993) Nuclei which lack a lamina accumulate karyophilic proteins and assemble a nuclear matrix. *J Cell Sci* **106**: 275–285

Kamat AK, Rocchi M, Smith DI and Miller OJ (1993) Lamin A/C gene and a related sequence map to human chromosomes 1q12.1-q23 and 10. *Cell Mol Genet* **19**: 203–208

Kaufmann SH, Mabry M, Jasti R and Shaper JH (1991) Differential expression of the NE lamins A and C in human lung cancer cell lines. *Cancer Res* **51**: 581–586

Lanoix J, Skup D, Collard JF and Raymond Y (1992) Regulation of the expression of lamins A and C is post-transcriptional in P19 embryonal carcinoma cells. *Biochem Biophs Res Comm* **3**: 1639–1644

Lebel S, Lampron C, Royal A and Raymond Y (1987) Lamins A and C appear during retinoic acid-induced differentiation of mouse embryonal carcinoma cells. *J Cell Biol* **105**: 1099–1104

Lehner CF, Stick R, Eppenberger HM and Nigg EA (1987) Differential expression of nuclear lamin proteins during chicken development, *J Cell Biol* **105**: 577–587

Lin H and Worman HJ (1993) Structural organization of the human gene encoding nuclear lamin A and nuclear lamin *C J Biol Chem* **268**: 16321–6

Lourim D and Limm JJ-C (1992) Expression of wild-type and nuclear localization deficient lamin A in chick myoblasts. *J Cell Sci* **123**: 501–512

Machiels BM, Broers JLV, Raymond Y, de Ley L, Kuijpers HJH, Caberg NEH and Ramaekers FCS (1995) Abnormal A-type lamin organization in a human lung carcinoma cell line. *Eur J Cell Biol* **67**: 328–335

Machiels BM, Zorenc AHG, Endert JM, Kuijpers HJH, van Eys GJJM, Ramaekers FCS and Broers JLV (1996) An alternative splice product of the lamin A/C gene lacks exon 10. *J Biol Chem* **271**: 9249–9253

Machiels BM, Ramaekers FCS, Helma JH, Kuijpers HJH, Groenewould JS, Oosterhuis JO and Looijenga LHJ (1997) Nuclear lamin expression in normal testis and testicular germ cell tumours of adolescents and adults. *J Cell Biochem* **62**: 275–289

Mittnacht S and Weinberg RA (1991) G1/S phosphorylation of the retinoblastoma protein is associated with an altered affinity for the nuclear compartment. *Cell* **3**: 381–393

Nigg EA (1989) The nuclear envelope. *Curr Opin Cell Biol* **1**: 435–440

Ozaki T, Saijo M, Murakami K, Enomoto H, Taya Y and Sakiyama S (1994) Complex formation between lamin A and the retinoblastoma gene product: identification of the domain on lamin A required for its interaction. *Oncogene* **9**: 2649–2653

Paulin-Levasseur M, Scherbarth A, Giese G, Roser K, Bohn W and Traub P (1989) Expression of vimentin and nuclear lamins during the in vitro differentiation of human promyelocytic leukemia cells HL-60. *Eur J Cell Biol* **50**: 453–461

Pugh GE, Coates PJ, Lane EB, Raymond Y and Quinlan RA (1997) Distinct nuclear assembly pathways for lamins A and C both lead to their increase during quiescence in Swiss 3T3 cells. *J Cell Science* **110**: 2483–2493

Quinlan R, Hutchison CJ and Lane EB (1995) Intermediate Filament Proteins. *Protein Profile* **2**(8)

Röber R-A, Weber K and Osborn M (1989) Differential timing of nuclear lamin A/C expression in the various organs of the mouse embryo and the young animal: a developmental study. Development **105**: 365–378

Rowlands DC, Bunce CM, Crocker J, Ayres JG, Johnson GD, Ling N and Brown G (1994) Expression of a NE protein recognised by the monoclonal antibody BU31 in lung tumours: relationship to Ki67 antigen expression. *J Pathol* **173**: 89–96

Schatten G, Maul GG, Schatten H, Chaly N, Simerly C, Balczon R and Brown DL (1995) Nuclear lamins and peripheral nuclear antigens during fertilisation and embryogenesis in mice and sea urchins. PNAS **82**: 4727–4731

Stewart M and Burke B (1987) teratocarcinoma stem cells and early mouse embryos contain only a single major lamin polypeptide closely resembling lamin B. Cell **51**: 383–392

Stick R and Hausen P (1985) Changes in the nuclear lamina composition during early development of *Xenopus laevis* Cell **41**: 191–200.

Templeton DJ, Park SH, Lanier L and Weinberg RA (1991) Nonfunctional mutants of the retinoblastoma protein are characterised by defects in phosphorylation, viral oncoprotein association, and nuclear tethering. Proc. Natl. Acad. Sci. USA **88**: 3033–3037

Vaughan AO, Whitfield WGF and Hutchison CJ (2000) Lamina function in nuclear envelope growth and DNA replication. Protoplasma 211, 1–7

Zhang C, Jenkins HE, Goldberg M, Allen T and Hutchison CJ (1996) Nuclear lamina and nuclear matrix organisation in sperm pronuclei assembled in *Xenopus* egg extract. *J Cell Sci*. **109**: 2276–2285

© 2001 Cancer Research Campaign
